# The Seventh Census, &c.

**Published:** 1855-03

**Authors:** John E. Sanborn


### IOWA MEDICAL JOURNAL.

VOL II. KEOKUK, IOWA, FEB. & MARCH, 1855. NO. 3.

### ORIGINAL COMMUNICATIONS.

### THE SEVENTH CENSUS, LOOKED AT PROFESSIONALLY.

BY PROF. JOHN E. SANBORN, M. D.

WE have spent a very pleasant hour in examining the "Compendium of the Seventh Census," an exceedingly interesting document of the House of Representatives, comprising 400 pages, and admirably compiled under the direction of J. D. B. DeBow, Superintendent of the U. S. Census. We confess to a strong liking for the volume. Though not strictly a rhetorical work, it abounds in *figures*, not dwelt upon by Blair or Whately. Forbidding as the long array of tables and statistics usually appears, and uninviting as may seem the hundreds of pages of solemn columns and figures, marshaled along like the rank and file of soldiers at a country muster, yet, when duly questioned, these mute military certainly tell to us a most eloquent and interesting story. Few studies are more full of interest than those which involve a wide survey of the human race, and contemplate its history, its progress, and its illimitable future : and so we propose to spend a little time with you, good reader of the Journal, in looking up some of the more interesting portions of the Census, particularly such as are connected with the pursuits and meditations of us medical men.

We say it almost every day, and yet we daily repeat it as a new thing, "how astonishing the growth of our country !" When we turn over the ample leaves, and glance at the stupendous statistics of our census, we must confess that this brother Jonathan, though now in the very flush of opening manhood, has certainly been pretty busy, thus far, and seems, in the volume before us, quite able to give a good account of what he has been doing. Our population is to-day about *nine* times what it was at the formation of the government—has increased nine fold in about seventy-five years, or doubled every eight years. States that had no census at all in 1830, now number over 350,000! A pleasant farm, which in 1825 was unnoticed, is now a city numbering some 75,000, having grown at the uniform rate of about 3,000 a year for twenty-five years!

It is intimated to us in various parts of the crowded volume before us, that some of the tables and statistics may not be entirely reliable, to the minutest extent, from a variety of causes: sometimes the forgetfulness of families respecting events which transpired within a year or two; sometimes the unwillingness of individuals to state the truth, especially concerning deaf, dumb, blind, or insane relatives, and particularly, says a friend at our elbow, concerning their own ages; oftentimes also in such a gigantic collection of individual facts, human errors will invariably creep in.

In a country, too, so large as ours, the migratory habits of quite a fraction of the population must always either destroy the original accuracy, or mar the permanency of the record. As the census stands, however, we may consider it a very reliable approximation to the truth, as a whole, even in the most difficult departments, while in others it is probably almost the absolute verity. In truth, the accuracy of our national census is probably greater than that of any nation on earth, owing to the greater general intelligence of our people, and their ability, and almost uniform willingness, to communicate the actual truth.

The total area of the United States, on the first day of January, 1854, is put down as 2,936,166 square miles; while the total population of the country is about twenty-five millions. This, by a ready calculation, would give about fifty acres to every human being in the Union.

The population of Iowa, for successive years, has been, in round numbers, as follows: in 1842, it was 58,000; in '45, 91,000; in '48, 142,000; in '52, 259,000; in '54, 349,000. The per centage of increase of the population of Iowa for the ten years from 1840 to 1850 is 345.85 per cent. This is exceeded by but one state in the Union, Wisconsin, whose growth for the same period is put down at the astonishing figure of 886.88 per cent.

With reference to the proportion of the sexes in the population of the Union, it seems that the ratio of females to males is at present exactly .95 per cent.; and this is less than it has ever been in the history of the country, being probably affected to some extent by the proportions of the sexes in the foreign immigration. At the periods of all the previous census, it has usually been a fraction over .96 per cent. In only one portion of the country, in happy New England, are the females in excess; and there, as we might infer, they always have had the majority, having been, at one census, 1820, as high as 103.46 per cent. Possibly the excess at that time was partly due to the effects of the war of 1812–14. In all other portions of the Union, males have always been in excess, this being generally greatest in the South-West, where it is about 100 males to 91 females. In the Territories and California there are nearly three times as many males as females.

With reference to the age of the white population, it is a matter of impressive interest, the immense preponderance of young persons. The age of 19 years nearly divides the whole population into halves. More than one half are under twenty years of age, about two fifths are between 20 and 50, and less than one fifth are over 50 years of age. This is "young America," indeed! Divide the whole population of the United States into thirds, and one third is under 10 years of age, another between 10 and 26, and the other one third over 26. That nearly one half of the people of this country should be under 19 years of age, and two thirds of the population under 26 years! In the year 1850 the number of persons of 100 years of age and upwards was 787. It is a little interesting to notice that of this number a large majority were females, they being 430, and males but 357—a result in confirmation of the established law respecting the longer life of females. During the first half of her life time the female is incident to more dangers than during the last; and when she has passed the age of fifty years, her chance for an old age is therefore better than that of a man, who is still subject to the effects of labor, anxiety and exposure. As we go down the scale of human life, how rapidly the number increases. Persons of the age of 100 and over, 787; between the ages of 90 and 100, more than 8,000; between 80 and 90, more than 65,000; and so downwards.

Of deaf and dumb persons, the number in the Union in 1850, is stated to be 9,136; of blind, 7,978; of the insane, 14,972; and of idiots, 14,257. In our own State, Iowa, there were, in 1840, deaf and dumb, 10; but in 1850 there were 59. Of blind, in 1840, there were three; but in 1850, 50. Of insane and idiotic, (classed together by that census,) there were, in 1840, only 7; but in 1850, we have, of insane, 42, and of idiots, the number written is 94. We think we have reason to assert, that this statement of insanity for Iowa is incorrect, being in fact quite too low, as the remarkable disproportion between the number of insane and idiots, might at once suggest. During the last year, the obvious errors of the census, in this respect, attracted the attention of Prof. McGugin, whose thorough familiarity with this subject is due to his having been, for many years, an able medical officer of the Ohio State Insane Asylum. For the purpose of eliciting correct information respecting this subject, he prepared printed circulars of enquiry, which were sent to the proper officers of every county in the State. Returns were received from only twelve, out of more than fifty settled counties in the State, and these twelve not the most populous. These twelve counties gave 37 insane, averaging three to a county. Even this proportion, extended to only the fifty more settled counties of the State, would give 150 insane, instead of 42. But, of course it will be seen that a portion of this excess must be due to the growth of the State, from 1850 to 1854. During these four years, the population of the State has almost exactly doubled. Double then the census figure of the insane in 1850, to adapt it to 1854, and it even then amounts to only 84, but little more than one-half the number estimated by Professor McGugin, and that too, in 1853, now more than a year ago. An interesting article, on this same subject, by the gentleman alluded to, will be remembered by the reader of this Journal, and may be found in the October No. of the last Vol.

We regret that we cannot pass by these statistics of the insane and idiotic of Iowa, without another comment. By the census, the number of idiots in Iowa is altogether out of proportion to that in other States, being more than double that of the insane. It would hardly seem probable that the enterprising families who form the chief population of so new a State, should bring with them to their new homes, so many individuals whose presence certainly could not be a source of pleasure to them: nor would the natural increase of the population during the brief age of the State, afford so large a proportion of these unfortunate objects of pity. A recent message, sent by Gov. Grimes to the Senate, also indicates a much less proportion. So too regarding the blind, of whom the census reports as we have seen, but 50. Mr. Bacon, Superintendent of the Blind Asylum, has published a list of 89 blind, personally known to him, and is of opinion that there are not less than some two hundred.

Some interesting statistics are found respecting the number of births in different portions of the country. The proportion or per centage of these varies widely, in the different States, depending upon various causes, the most prominent of which are the social institutions, or the want of them, as in Utah, the health of the different States, and the relative age of the residents. The per centage of births varies from 3.80 of Utah, down to .29 of California. The States favored with the largest proportion of births, are as follows, arranged in the order of their per centage: Utah, Wisconsin, Arkansas, Missouri, Indiana, Iowa, Illinois, Kentucky, Texas and Tennessee; all, as will at once be seen, Western States. Those States afflicted with the smallest proportion of births, are as follows, in order, from the lowest up: viz. California, New Hampshire, Connecticut and Vermont, all fairly reckoned Eastern States. In this computation, the two extreme States, Utah and California, may, for manifest reasons, be fairly set aside, as they are really exceptional, and then it will be readily apparent that all the prolific States are Western, and all the barren ones Eastern; a result probably in part accounted for by the great immigration of young persons to the West, from the North-eastern States, which has left the "old folks at home." The general ratio for all portions of the country shows 2.75 per cent. of births to every hundred free persons or one birth to every 36 persons.

A medical writer states, that nine months after the visit of the cholera to Philadelphia, there was a remarkable diminution in the proportion of male births, confirming the conclusion that disease, epidemics, exhausting labor, poor diet, bad ventilation, intemperance and other such social evils, generally diminish the number of male births. It seems well established that all measures which promote the health and welfare of a population, not only increase the capacity for profitable labor, but also tend to promote the multiplication of the male sex. Thus, in England, the excess of male births is only 5 per cent.; in France and Prussia, 6 per cent.; in Philadelphia, 7 per cent., and in Kentucky, 12½ per cent. In the cities of Massachusetts, it is but about 6 per cent., but in the agricultural districts, it reaches 9 per cent. It would be an interesting point of enquiry, whether the conclusion alluded to, with reference to the preponderance of male births under conditions of greater parental health, vigor and vitality, would not be borne out by a comparison of any number of families. Let any person contrast those families, within his memory, whose children are mostly males, and see if there be not sufficient grounds in the physique of the parents to account for the difference.

The statistics respecting the localities and proportions of marriages have somewhat less of interest, as many marry at a distance from the State of their residence. The percentage of marriages ranges from Utah, the highest, which is 3.56, down to unfortunate Delaware, which can report only a fractional .63. The largest proportions, ranging from the highest in order down, are in Utah, New Mexico, Texas, Oregon, Arkansas, Indiana, and Missouri; the smallest are Delaware, Minnesota, South Carolina, Maryland, District Columbia, Maine, Connecticut and Florida. The ratio of marriages is very nearly one person married to every two hundred of population, varying between the States, from one to 316, as in Delaware, to one to 102, as in Massachusetts. The conclusion of the compiler of the compendium is, that the returns of the census are not in this respect fully reliable, and that probably the actual proportion in the United States cannot differ much from that of Massachusetts, and is no doubt even larger than that.

In regard to the very important subject of necrology, or the statistics of deaths, the returns of the census are unfortunately far too deficient. The States showing, according to the returns, the largest proportions of death are put down as follows, and in the order of their unfortunate precedence: Louisiana, Utah, Massachusetts, Maryland, District of Columbia, Missouri, Connecticut and Rhode Island. In all these States the ratio of deaths to the whole population ranges at various figures from Rhode Island, which is 1.52 per cent. up to Louisiana, which is highest, at 2.23; all these eight States having over 1.50 per cent. The favored States which indicate the least number of deaths, or the smallest proportion, are as follows, in order, from the least upwards: Oregon, Minnesota, Georgia, Wisconsin and California: these ranging from Oregon, the smallest, which is only a fractional .35 per cent. to California, which is .98 per cent.; all below 1 per cent. Directly in order after these last States, so very prominent for their apparent exemption from death, come in as next most healthy, a phalanx of southern States: viz. Florida, North and South Carolina, Alabama, Tennessee and Mississippi, these being but a trifle over one per cent.

We cannot but suspect that the returns indicating so very small a proportion of deaths in Oregon, one to nearly three hundred persons, and in Minnesota, one to two hundred, must be incorrect, and for this obvious reason. It is at once apparent that in these new States there are comparatively few permanent settled families, such as would be likely to remember and report all the deaths that have occurred within the year prior to June, 1850; but, on the other hand, that the population is largely composed of strangers, transitory and single persons, of unsettled habits, many of whom may die and leave no surviving friends, whose faithful memory shall be their record. There seems, however, no reason to doubt that the States alluded to are probably the most healthy in the Union.

The general proportion of deaths to the population, for the whole Union, the average of all the States, is put down at 1.39 per cent., or, in other words, one death to every seventy-two persons; the two extremes being in Louisiana, one to every 44, and in Oregon, one to every 283. The Massachusetts State Census, taken every year, shows, for that State, during three years, a general average of one death to every 53 persons. The final summary may be stated as follows: That the actual number of deaths in the whole Union, for 1850, reckoning it a sickly year, could not have fallen short of one in every fifty persons, an estimate which would make the total number of deaths over 463,000.

A calculation has been made of the mortality of our most prominent cities and with the following results, all probably reliable for a long term of years, except New Orleans, where the estimate was made during only a few and those unusually sickly seasons. In Charleston, the deaths are estimated as one to 48; in Boston, one to 46; in Philadelphia, one to 45; Baltimore, one to 43; Cincinnati, one to 35; New York, one to 34; New Orleans, one to 19.

The per centage of deaths in some American and English cities runs as follows: In New York City, 2.55 per cent; Baltimore, 2.49; Charleston, 2.48; Boston, 2.45; Lowell, 2.11; in the State of Massachusetts, 1.59. In twelve counties of England, 1.93 per cent.; in twenty-six cities of England, 2.72; in London, 2.53; in Liverpool, 3.34; in Manchester, 3.48.

Dr. Barton, states that the proportion dying from all pulmonary diseases is, in Philadelphia, 28.57 per cent.; in New York City, 28.08 per cent.; in Havanna, 25.07 per cent.; in Boston, 23.97; Baltimore, 23.33; Charleston, 22.73; City of Mexico, 16.76; Norfolk, Va., 12.78; New Orleans, 13.87 per cent. An expanded view over so wide a range of facts, often enables us to correct misapprehensions respecting the relative health of different cities and localities, into which we had gradually, and half unconsciously allowed ourselves to fall.

In lieu of any published tables in the census returns, of the proportions of deaths at different ages, we beg leave to offer the following table of the centennial proportions of deaths at different ages, that occurred in England during seven years. The main features will apply closely to our own county, and present some points of interest:

| | | |
|---|---|---|
| Deaths under 5 years of age, - - | 39.66 | per cent. |
| Of 5 years, and under 10 - - - | 4.99 | " " |
| " 10 " " " 15 - - | 2.62 | " " |
| " 15 " " " 25 - - - | 7.40 | " " |
| " 25 " " " 35 - - | 6.97 | " " |
| " 35 " " " 45 - - - | 6.35 | " " |
| " 45 " " " 55 - - | 6.07 | " " |
| " 55 " " " 65 - - - | 7.06 | " " |
| " 65 " " " 75 - - | 8.66 | " " |
| " 75 " " " 85 - - - | 7.52 | " " |
| " 85 " " " 95 - - | 2.50 | " " |
| " 95 and upwards, - - - | .20 | " " |
| | 100.00. | |

A table somewhat similiar to this, is presented of the City of Boston, but as the per centage is calculated upon a different basis, its citation would not answer the purpose of contrast.

It may be a matter of interesting local calculation to learn that Iowa is surpassed, in the important matter of the proportion of births, by only four States; while, in the equally *vital* consideration of death, there are but nine States having a less per centage. The combined testimony of these two facts is so favorable and unequivocal as to need no comment. An extended table is given, in the compendium, of the occupation of the male population of the United States, over fifteen years of age. From this table it appears that the number of physicians in the country, is 40,564. We are not informed whether or not this includes every color and form of the great genus "Physic," but we may be allowed to hope it does, although a distinction should be made. At any rate, the number given makes the proportion of medical men about one to every 650 of the population. This is very nearly the proportion indicated by the State Census of Massachusetts, and of some of the other States. If this should appear to any one a large proportion, there may be some consolation in the fact that the number of undertakers is stated to be only 495, or one undertaker to about eighty-five physicians. The whole number of lawyers is stated as 23,939, and of clergymen as 26,842.

From a table giving the proportions of the leading occupations in the great geographical divisions of the Union, it appears, as the first great fact, that the learned professions, if we may rely on the accuracy of the returns, have filled up wonderfully since 1840. To every thousand of population, there were in 1840, only 3.81 per cent. of the learned; but the year 1850 finds a proportion of 9.23 per cent. These are distributed as follows: The southern States, having of course the smallest share, 1.17; the southwestern, 1.21; New England States, 1.22; middle States, 2.79; the northwest, 2.82, the largest proportion. Ranked as slave and free States, the contrast is what every one would expect; the South has 3.30 per cent. and the North 5.93.

Among the educational statistics, which comprise many very interesting tables, is an enumeration of the various colleges in the Union. The number of medical schools is put down at 36. This statement would seem to be a little unfortunate, as we have before us, in a medical publication, a list of 42 medical colleges in the United States. The error may, however, have occurred from the fact that some are in a state of coma, sufficient to warrant their omission from the enumeration. The number of medical colleges in the Union is, by the census 37, of professors 253, and of students of medicine 5,025, or about one-eighth the number of physicians in the Union. Contrast with this proportion, the number of theological students, 1,351, which is about one-twentieth the number of clergymen in the country, or the number of law students, 532, less than one-fortieth the number of lawyers in the Union, and, if the census has a shadow of truth, we are quite willing, as physicians, to accept the conclusion indicated, even after making a liberal allowance for a large proportion of private students.

We should be pleased to continue these running selections and comparisons to a greater length, for the field is ample and inviting; and many interesting and sometimes unexpected results are often arrived at, by a careful comparison of the materials so liberally furnished by the Census Reports; but we fear we have already taken liberties with the patience of the reader.

We cannot close these remarks without expressing an earnest sense of the great and almost criminal neglect of opportunity, if not of duty, by the officers of our several State Census, in omitting to collect and publish statistics respecting the birth, disease and death of every individual in the country. It needs but a well organized system of action, that this may be done promptly and accurately. In no way can society be so signally benefited, as by a more general enlightenment, upon this neglected topic. It should be made, by law, the duty of every physician or attendant, to report to a proper officer, every birth, and every death, with its cause and the age at death. Careful collections of these facts should be made by legally appointed officers in every city, town and county of every State in the Union; and these reports, when collected, combined and compared, would furnish a most interesting and valuable means of medical and sanitary investigation and improvement. A great crime of neglect is committed, as long as these duties hang upon our hands, and demand our attention.

But, even if no means are being taken for the collection of vital statistics in future, we may well suppose that those already in the hands of the Census Department, might be published. We have spoken of the signal and remarkable barrenness of the census, in respect to this matter; and it is rather matter for surprise than consolation to read, in the compendium, such a sentence as this: "Should the mortality statistics of the census be printed, (and they have been asked for by medical men, societies, and associations, in every part of the Union,) some very useful deductions could be made from them." Well, why are they not printed? Why are we not allowed the benefit of these useful deductions? Are the united wishes of the medical men of every part of the Union of no significance? Then, why are the mortality tables not published? Where are they? Packed away in some dusty corner at Washington, useless and unnoticed. And who shall tell us why they are not brought to light? why tens of thousands are spent to diffuse the flatulent rhetoric of Washington, while the country has demanded the publication of its vital statistics, and suffers for the want of it? We send up our voice in favor of the publication of these mortality tables, and in favor of a complete and efficient legal collection of the full vital statistics of every portion of the Union.

